# Midazolam for the Successful Treatment of Refractory Spinal-Anesthesia-Associated Hypothermia After Cesarean Delivery

**DOI:** 10.7759/cureus.39492

**Published:** 2023-05-25

**Authors:** Alexander M DeLeon, Samantha Lu, Ian Gaston, Alexander G Samworth, Carmen Lopez, Jason Farrer

**Affiliations:** 1 Anesthesiology, Northwestern Memorial Hospital, Chicago, USA; 2 Obstetric Anesthesia, Northwestern Memorial Hospital, Chicago, USA; 3 Anesthesiology, Northwestern University Feinberg School of Medicine, Chicago, USA

**Keywords:** post-anesthesia care, temperature regulation, cesarean delivery, hypothermia, midazolam

## Abstract

Cesarean deliveries receiving spinal anesthesia with intrathecal morphine are associated with post-operative hypothermia. Lorazepam has been proposed as a reversal agent for treating intrathecal morphine-associated post-cesarean hypothermia. Midazolam is a benzodiazepine familiar to most anesthesia providers and is frequently administered in the perioperative period. We present a post-cesarean delivery spinal anesthesia-associated hypothermia patient successfully treated with intravenous midazolam.

## Introduction

Refractory post-cesarean delivery spinal anesthesia-associated hypothermia has been reported with an average temperature drop of >1.0 °C [1-4]. Hui et al. [4] performed a randomized, controlled, double-blinded trial linking intrathecal morphine to post-cesarean delivery hypothermia. Hess et al. [1] reported using lorazepam to reverse intrathecal morphine-associated hypothermia in the setting of cesarean delivery. The use of midazolam in this setting has not been reported in the literature.

We report the first case of refractory hypothermia successfully reversed with intravenous midazolam in a patient who received intrathecal morphine for cesarean delivery.

## Case presentation

A 42-year-old, G1P0 at 39.1 weeks, presented for an elective cesarean delivery. The patient was recently diagnosed with a left atrial mass, and thus her cardiologist recommended avoiding Valsalva maneuvers associated with vaginal birth. Her recent echocardiogram demonstrated normal systolic function with a visually estimated ejection fraction of 55%. The mass was visualized and attached to the left side of the interatrial septum. The mass measured 1.5 cm × 0.9 cm. The echocardiogram revealed no other pathology.

Her height was 5'2", and her weight was 63.8 kg (BMI 25.72 kg/m^2^). Her other labs and vital signs were all within normal limits, and her physical exam was non-contributory. At 12:23 on the day of surgery, the patient received a spinal anesthetic consisting of 12 mg of hyperbaric spinal bupivacaine, 15 mcg of fentanyl, and 150 mcg of preservative-free intrathecal morphine. A phenylephrine infusion was used post-spinal anesthetic, per protocol. Other medications received intraoperatively included 30 mg of ketorolac, 4 mg of dexamethasone, and 4 mg of ondansetron. Post-delivery, the patient received oxytocin as an infusion at 18 units per hour. The intraoperative fluid administration consisted of 1.7 L of lactated ringers. The estimated blood loss was 625 mL, and the urine output was 400 mL.

The patient's surgical procedure concluded, and she arrived in the recovery unit at 13:09. The patient's sensory level on arrival in the recovery room was T3 bilaterally to pinprick. The patient's temperature was undetectably low by the oral, scalp, or axillary thermometer. A forced-air full-body warming blanket (Bair Hugger, Model 775, 3M, St. Paul, Minnesota) was instituted at the highest temperature (43 °C). The patient did not report subjective hypothermia symptoms.

At 13:51, the patient's temperature read 34.6 °C, and the forced air-warming blanket was continued. The patient was still asymptomatic. The repeated oral temperature at 14:21, after 30 minutes, was 34.9 °C despite having received an hour of active full-body forced-air warming. Her spinal pinprick sensory level at this time was T6 bilaterally. The patient did not receive significant intravenous fluids as she showed no evidence of hypovolemia.

At 14:25, the anesthesia attending administered 1.0 mg of intravenous midazolam due to the lack of significant improvement despite the warming blanket. Twenty minutes later, her oral temperature was measured at 36.3 °C. The spinal sensory level did not change significantly over the 20 minutes between the midazolam dose and the temperature measurement of 36.3 °C. The remainder of her hospitalization and recovery were uneventful, with her maintaining a temperature of >36.0 °C throughout.

## Discussion

The association between intrathecal morphine and persistent hypothermia post-cesarean delivery after spinal anesthesia has been reported in numerous case reports [2-4]. A randomized, double-blind, controlled study by Hui et al. [4] supported intrathecal morphine as the likely cause of persistent hypothermia post-cesarean section. Their group found a greater decrease in temperature in the group receiving morphine versus the control group (p = 0.01), with the nadir in temperature lasting approximately 10 minutes longer with the morphine group (p = 0.047).

Hess et al. [1] reported a case series of 14 patients with persistent hypothermia post-cesarean delivery under spinal anesthesia who received intrathecal morphine. Patients in their report had measured temperatures less than 35.8 °C. Hess et al. [1] reported that 4 of the 14 patients who did not receive lorazepam were continuously hypothermic for six hours. The 10 of 14 patients who received lorazepam experienced near immediate cessation of symptoms and resolution of hypothermia, with a statistically significant (p < 0.05) temperature difference until 180 minutes post-cesarean section.

Assuming morphine and lorazepam are, in fact, implicated in hypothermia and reversal. In that case, the mechanism for their respective effects has been proposed to involve the thermoregulatory centers of the central nervous system [1]. Midazolam was hypothesized at our institution to have a similar central effect to lorazepam. Given midazolam's familiarity among anesthesia providers, it would be the preferred hypothermia reversal agent if shown to be effective.

The rapid resolution of hypothermia after the administration of midazolam suggests a positive response. The resolution time was similar to that reported by Hess et al. [1], who found a statistically significant difference between patients receiving lorazepam and those continuing with conservative warming measures within 30 minutes.

The main limitation of this report is that case reports have a low sample size, thus limiting the generalizability of the findings. We report the first case of the use of intravenous midazolam to treat persistent intrathecal morphine-associated hypothermia after cesarean delivery.

## Conclusions

Despite the use of forced air-warming blankets after cesarean delivery, persistent hypothermia is a clinical challenge facing obstetric anesthesiologists. The causation of hypothermia by intrathecal morphine is supported by randomized controlled trials. The use of lorazepam has been reported to reverse hypothermia. We present a case of refractory post-cesarean delivery hypothermia after spinal anesthesia with intrathecal morphine that responded positively to a single dose of intravenous midazolam.
